# Spillover can limit accurate signal quantification in MPI

**DOI:** 10.1038/s44303-025-00084-0

**Published:** 2025-05-06

**Authors:** Ali Shakeri-Zadeh, Shreyas Kuddannaya, Adnan Bibic, Jeff W. M. Bulte

**Affiliations:** 1 The Russell H. Morgan Department of Radiology and Radiological Science, Division of MR Research, The Johns Hopkins University School of Medicine, Baltimore, MD USA; 2 Cellular Imaging Section and Vascular Biology Program, Institute for Cell Engineering, The Johns Hopkins University School of Medicine, Baltimore, MD USA; 3 F.M. Kirby Research Center for Functional Brain Imaging, Kennedy Krieger Inc., Baltimore, MD USA; 4 Department of Oncology, Johns Hopkins University School of Medicine, Baltimore, MD USA; 5 Department of Chemical & Biomolecular Engineering, Johns Hopkins University Whiting School of Engineering, Baltimore, MD USA; 6 Department of Biomedical Engineering, The Johns Hopkins University, Baltimore, MD USA

**Keywords:** Engineering, Nanoscience and technology, Physics

## Abstract

Accurate quantification of the magnetic particle imaging (MPI) signal in vivo remains a significant technical challenge. We assessed the “spillover effect”, defined as leakage of signal from adjacent areas within a region of interest, within a field of view containing multiple hot spots, a scenario frequently encountered in vivo after systemic administration of a magnetic tracer. Using custom-designed phantom and in vivo mouse studies we determined the impact of fiducial positioning, iron content, and the iron concentration ratios within those hot spots, as well as the suitability of four different MPI scan modes for accurate signal quantification. Adjustment of the specific “target-to-fiducial distance (TFD)” and “target-to-fiducial Fe concentration ratios (TFCR)” significantly reduced the spillover effect. It’s implementation to mitigate spillover effects will increase the accuracy of MPI for in vivo magnetic tracer quantification.

## Introduction

Magnetic particle imaging (MPI) has gained momentum as an emerging imaging modality due to its high sensitivity and ability to quantitatively track magnetic nanoparticles (MNPs) including superparamagnetic iron oxide (SPIO) in vivo. These attributes make MPI an invaluable tool for overall pharmacokinetic profiling of in vivo SPIO distribution^1^ and specific applications including cell tracking^2–6^, tracking of extracellular vesicles (EVs)^7,8^, image-guided hyperthermia^9–12^, and vascular imaging^13,14^, to name a few. Despite its promise, MPI faces significant technical challenges, particularly in accurately quantifying signals. In one study^15^, the importance of the selection of the region of interest (ROI) was emphasized, highlighting that defining the ROI cutoff can significantly affect the accuracy of iron quantification. MPI noise and background signal can also adversely affect accurate signal quantification and potentially hinder clinical translation by impacting sensitivity^16^. Moreover, studies show that the dynamic magnetic behavior of MPI tracers, such as ferucarbotran^17^, can be altered at higher concentrations, also affecting MPI signal quantification. Building upon on our recent MPI experience from conducting over one hundred in vivo and ex vivo experiments^18–21^, we have identified another crucial aspect of MPI signal quantification which we term “the spillover effect”. This effect in MPI is akin to the partial volume effect (PVE) in nuclear imaging^22^ and is also referred to by other MPI investigators as “shine-through”^23^. The spillover effect may occur when magnetic signals from an ROI leak into adjacent areas, potentially compromising the accuracy of signal quantification in those regions. This phenomenon complicates the accurate quantification of MNPs, especially in scenarios involving multiple hot spots within the same field of view (FOV)^24–26^. Such situations are common in dynamic in vivo studies, where either MNPs or MNP-labeled cells/EVs may distribute across nearby organs, leading to overlapping signals and potential inaccuracies.

In a typical quantitative MPI scenario, fiducials containing a known amount of MNPs are used for signal calibration and quantification^27,28^. Two predominant methods are used for this purpose^28^: (1) placing fiducials within the same FOV as the study subject, followed by regression analysis of the calibration curve to estimate MNP concentration in observed hot spots; and (2) scanning fiducials and the subject independently to eliminate signal interference within the FOV. The latter method is preferred for enhancing accuracy but is often impractical for fast dynamic in vivo studies, especially when multiple hot spots across various organs coexist. For instance, in the realm of cell therapy for brain disease, intra-arterial injections of MNP-labeled stem cells via internal carotid artery catheterization have shown promise as an effective method for global cell delivery^29,30^. However, this delivery approach often results in the distribution of cells in multiple organs such as the brain, lungs, and liver wherein accurate quantification of MPI signals becomes challenging due to overlapping signals in different hotspots^20^. Inter-organ analysis or comparing signals between distinct and spatially separated organs (e.g., brain versus liver) would be straightforward in human MPI due to the farther proximity of organs and structures. However, small animal imaging or intra-organ analysis in human imaging present greater challenges, where higher MPI spatial resolution is essential to accurately differentiate closely situated tissue structures or lesions.

Although a shine-through or spillover resolution of <3.5 mm has been reported to identify two adjacent sources using a Momentum MPI scanner^23^, we show here that this resolution depends not only on the distance between the sources and the MPI scan mode, but also on the concentration ratio of sources. We conducted a series of in vitro phantom studies to determine an optimal distance for fiducial placement that would minimize signal interference during MPI studies, ensuring accurate signal quantification. We also assessed whether different scan modes (standard, high resolution (HR), high sensitivity (HS) and high concentration (HC)) may show different spillover effects. In vivo, dynamic MPI studies in Rag2 mice were performed after systemic injections of MNPs at varying doses followed by analysis of the fiducial-organ (liver and spleen) spillover effect. We show here that optimizing the target-to-fiducial distance (TFD) and the target-to-fiducial iron concentration ratio (TFCR) can effectively minimize spillover in different MPI scan modes, potentially enhancing the accuracy of MPI in quantifying magnetic tracers in vitro and in vivo.

## Results

### In vitro phantom studies

**Fig. 1:**
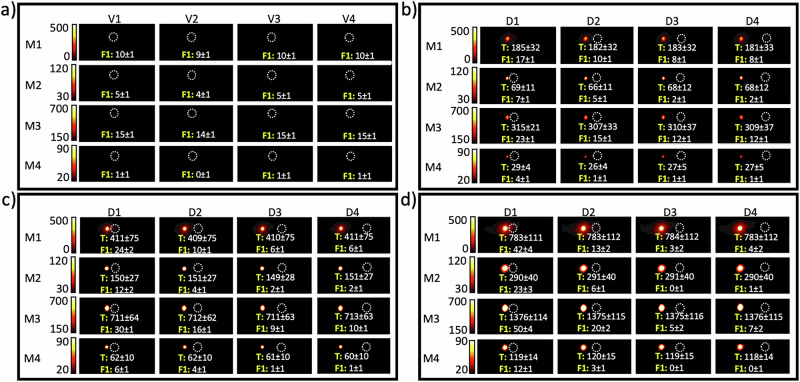
MPI signal quantification of Fiducial 1 (F1, 10 µg Fe/ml) for various target Fe concentrations, distances, and scan modes. **a** MPI signals of four vials (V1–V4) for the F1 set obtained with the four different scan modes (M1–4). MPI signal of F1 for different distances (D1–D4) from the target (T), with target Fe concentrations of **b** 200, **c** 400, and **d** 800 µg Fe/ml. Numbers represent the measured signal intensity for each tube. The values of TFCR featured in **b**–**d** are 20:1, 40:1, and 80:1, respectively. Numbers represent the mean signal value ± standard deviation of each hotspot/annotation. Empty dashed circles represent the location of the tubes without apparent MPI. M1 = standard; M2 = high resolution; M3 = high sensitivity; and M4 = high concentration mode. D1 = 10, D2 = 20, D3 = 30 and D4 = 40 mm.

**Fig. 2:**
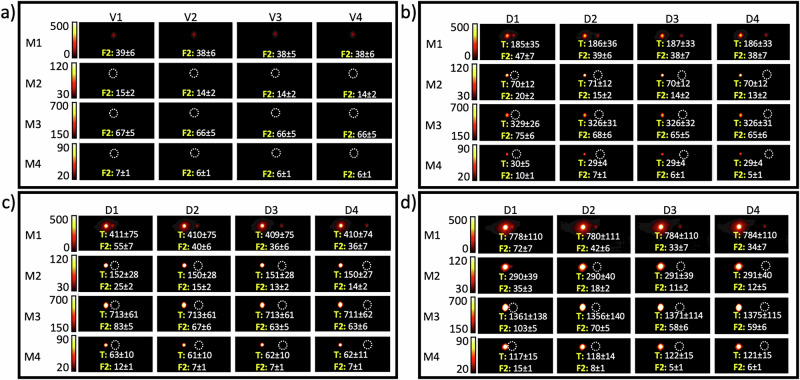
MPI signal quantification of Fiducial 2 (F2, 40 µg/ml) for various target Fe concentrations, distances, and scan modes. **a** MPI signals of four vials (V1–V4) for the F2 set obtained with the four different scan modes (M1–4). MPI signal of F2 for different distances (D1–D4) from the target (T), with target Fe concentrations of **b** 200 µg/ml, **c** 400 µg/ml, and **d** 800 µg/ml. Numbers represent the measured signal intensity for each tube. The values of TFCR featured in **b**–**d** are 5:1, 10:1, and 20:1, respectively. Numbers represent the mean value ± standard deviation of each hotspot/annotation. Empty dashed circles represent the location of the tubes without apparent MPI. M1 = standard; M2 = high resolution; M3 = high sensitivity; and M4 = high concentration mode. D1 = 10, D2 = 20, D3 = 30 and D4 = 40 mm.

**Fig. 3:**
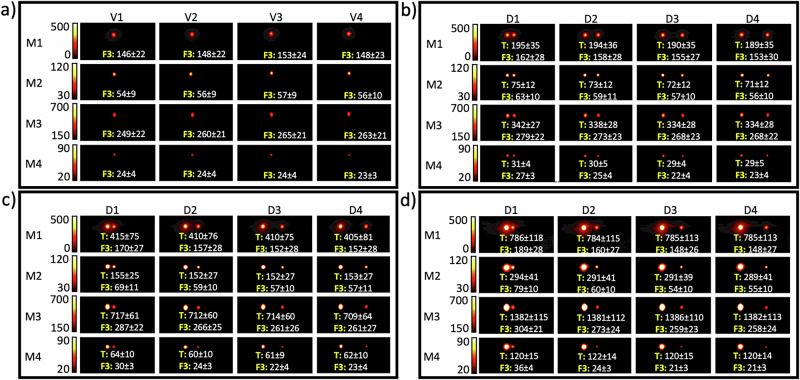
MPI signal quantification of Fiducial 3 (F3, 160 µg/ml) for various target Fe concentrations, distances, and scan modes. **a** MPI signals of four vials (V1–V4) for the F3 set obtained with the four different scan modes (M1–4). MPI signal of F3 for different distances (D1–D4) from the target (T), with target Fe concentrations of **b** 200 µg/ml, **c** 400 µg/ml, and **d** 800 µg/ml. Numbers represent the measured signal intensity for each tube. The values of TFCR featured in **b**–**d** are 1.25:1, 2.5:1, and 5:1, respectively. Numbers represent the mean value ± standard deviation of each hotspot/annotation. Empty dashed circles represent the location of the tubes without apparent MPI. M1 = standard; M2 = high resolution; M3 = high sensitivity; and M4 = high concentration mode. D1 = 10, D2 = 20, D3 = 30 and D4 = 40 mm.

Figures 1–3 show MPI signal quantification for three different fiducials, labeled F1, F2, and F3, imaged under different conditions using the four scan modes and various spacing distances and target Fe concentrations.

### Standard scan mode

For the standard scan mode, only F2 and F3 were visible, either when scanned alone within their own FOV or together with the target tube within the same FOV. F1 could be clearly distinguished when it was positioned 10 mm away from a target concentration of 200 µg Fe/ml, but its visibility diminished as the distance increased. As shown in Fig. 1, when F1 was positioned 10 mm from the target, the MPI signal increased by 70%, 140%, and 320% for target concentrations of 200, 400, and 800 µg Fe/ml, respectively. The MPI signal for F2 increased by 24%, 45%, and 89% at the same separation distance from the target (10 mm) with these respective Fe concentrations (Fig. 2). Figure 3 shows that for F3, at 10 mm distance, the MPI signal increase was 9%, 15%, and 28% for the corresponding target Fe concentrations. The increased signal in the fiducials due to their closeness to a high Fe concentration target clearly demonstrates a spillover effect, which tends to be more pronounced when the TFCR is higher, especially when the fiducial is in closer proximity to the target. These data obtained in standard scan mode suggest that a distance of 20 mm may be sufficient to avoid significant spillover effects, except when the TFCR reaches 80:1, which leads to a 30% increase in fiducial signal intensity (Fig. 1d). At Fe concentration ratios above 10:1, there was a notable signal reduction at distances of 30 and 40 mm, which became more pronounced as the TFCR was raised from 20:1 to 80:1. These findings could be reproduced in three independent repeats. For enhanced accuracy in standard mode MPI signal quantification, it appears that maintaining a TFCR ≤ 10:1 and a TFD ≥ 20 mm may be safe for proper signal quantification. Our results show that the visibility of fiducials was affected not only by the TFCR and TFD but also by the absolute iron content of the tubes. Despite an identical Fe concentration ratio (20:1) and distance (10 mm) for the samples shown in Figs. 1b and 2d, a higher iron content in tubes led to greater visibility of each tube. Hence TFCR, TFD, and scan mode are not the only factors influencing the visibility of an MPI spot; the absolute amount of iron content also plays a significant role for the spillover effect. A more concentrated target tube enhanced the visibility of the fiducial tube, even though the TFCR, TFD, and scan mode remained the same.

### High resolution scan mode

For the HR scan mode, F3 was visible at all tested distances from the target, demonstrating consistent visibility. Conversely, F2 was visible only when positioned 10 mm from the target, with a TFCR of 20:1 (Fig. 2d). Interestingly, despite F1 being subjected to a same Fe concentration ratio of 20:1 and a distance of 10 mm, it remained invisible in HR scan mode (Fig. 1b). This finding underscores that the iron content, distance, and concentration ratio all have an intrinsic effect on fiducial visibility using HR scan mode MPI. When F1 was positioned 10 mm from the target, the MPI signal increased by 40%, 140%, and 360% for target Fe concentrations of 200, 400 and 800 µg Fe ml, respectively (Fig. 1). However, the MPI signal for F2 increased by 42%, 78%, and 150% at the same distance from the target for the same respective Fe concentrations (Fig. 2). Figure 3 shows that for F3, at a 10 mm distance, the MPI signal increase was 12%, 23%, and 41% for these corresponding target Fe concentrations. Hence, similar to standard scan mode, an increased signal in the fiducials due to their closeness to a high Fe concentration target was seen for the HR scan mode, with the spillover effect being more pronounced in HR compared to standard mode. An exception was noted with F1 at a TFCR of 20:1, where the spillover effect was less pronounced compared to the same conditions in standard mode. This suggests that while the HR scan mode generally enhances spillover, a careful selection of the Fe concentration ratio and iron content—particularly ≤20 µg—may reduce the severity of the spillover effect. Our data suggest that a 20 mm separation between target and fiducial can mitigate spillover effects in HR scanning mode. However, at a high TFCR ratio of 80:1 (Fig. 1d), or with ratios ≥20 using increased iron content (Fig. 2d), we observed a 20% increase in fiducial signal intensity, indicating significant spillover under these conditions. Similar to standard mode, at a TFCR exceeding 10:1, there was also a notable signal reduction at distances of 30 and 40 mm. This effect intensified as the TFCR increased from 20:1 to 80:1. Hence, for proper MPI signal quantification in HR mode, a TFCR ≤ 10:1 and a TFD ≥ 20 mm appears appropriate.

### High sensitivity scan mode

Only F3 and not F1 or F2 was visible at all tested distances from the target. This suggests that a TFCR value ≤ 5:1 is necessary for a fiducial to be visible with HS-MPI scanning. Crucially, the iron content should be considered along with a TFCR of 5:1. At this ratio, F3 was visible in HS-MPI scans (Fig. 3d), while F2 remained invisible (Fig. 2b). This emphasizes the interplay between iron content and concentration ratio for fiducial visibility in HS-MPI scanning mode. As shown in Fig. 1, when F1 was positioned 10 mm from the target, the MPI signal increased by 53%, 100%, and 230% for target Fe concentrations of 200, 400 and 800 µg Fe/ml, respectively. Figure 2 indicates that the MPI signal for F2 increased by 13%, 25%, and 56% at the same distance from targets with these respective concentrations. Figure 3 shows that for F3, at a 10 mm distance, the MPI signal increase was 7%, 8%, and 14% for the corresponding target Fe concentrations. As observed in standard and HR scans, higher TFCR values amplify spillover effects in HS-MPI scans as well. However, HS-MPI scans generally showed a weaker spillover effect compared to standard and HR scan modes. Similar to standard and HR modes, our analysis indicates that maintaining a 20 mm separation between target and fiducial can generally mitigate spillover effects in HS-MPI scanning. However, when a high TFCR exists, as seen in Fig. 3d (ratio of 80:1), a significant spillover effect was observed for D2 with a 33% increase in fiducial signal intensity. Similar to the standard and HR modes, at a TFCR exceeding 10:1, there was also a notable signal reduction at distances of 30 and 40 mm. This effect intensified as the TFCR increased from 20:1 to 80:1. For enhanced accuracy in HS-MPI signal quantification, it is recommended to maintain values of TFCR ≤ 5:1 and TFD ≥ 20 mm.

### High Concentration scan mode

F1 and F2 remained not visible in HC scan mode as well. Similar to the other three scan modes, F3 was visible at all tested distances from the target. This suggests that a TFCR ≤ 5:1 is necessary for a fiducial to be visible within HC-MPI scanning. However, similar to HS mode, at a TFCR of 5:1, F3 was visible in HC-MPI scans (Fig. 3d), while F2 remained invisible (Fig. 2b). Figure 1 suggests that 1 µg of iron is not a suitable choice as a fiducial in an HC-MPI scanning. This is because the signal intensity from this concentration is not significantly different from background noise, rendering it undetectable and offering minimal value for quantification. However, when F1 was positioned 10 mm from the target (Fig. 1), the HC-MPI signal increased by 300%, 500%, and 1100% for target Fe concentrations of 200, 400 and 800 µg Fe ml, respectively. Figure 2 indicates that the HC-MPI signal for F2 increased by 66%, 100%, and 150% at the same distance from targets with these respective concentrations. Figure 3 shows that for F3, at a 10 mm distance, the MPI signal increase was 12.5%, 25%, and 50% for the corresponding target Fe concentrations. As observed in other scan modes, higher TFCR values enhance spillover effects in HC-MPI scanning. However, HC-MPI scans generally showed a stronger spillover effect compared to other three MPI scan modes. Similar to other scan modes, our analysis indicates that maintaining a 20 mm separation between target and fiducial can generally mitigate spillover effects in HC-MPI scanning if appropriate amount of iron is chosen for the fiducial (>1 ug Fe). However, under specific conditions of a high TFCR, as seen in Fig. 2d (ratio of 20:1), a significant spillover effect was observed for D2 by a 33% increase in fiducial signal intensity. Unlike other scan modes, a TFCR ≤ 5:1 resulted in a slight signal reduction at distances of 30 and 40 mm. This signal reduction was less pronounced compared to what was seen for other scan modes at the same distances when the TFCR was exceeding 10:1. For enhanced accuracy in HC-MPI signal quantification, it may be safe to maintain a TFCR ≥ 5:1 and a TFD ≥ 20 mm. Table 1 summarizes our recommendations for accurate MPI signal quantification when ferucarbotran is used as MPI tracer. Other MPI tracers, chemically engineered for obtaining higher resolution^31^, can be further studies to determine whether they allow for smaller distances or larger concentration ratios.

**Table 1:** Summary of TFCR and TFD recommendations for accurate signal quantification using the four MPI scan modes

MPI scan mode	TFCR recommendation (ratio)	TFD recommendation (mm)
Standard	≤10:1	≥20
High resolution	≤10:1	≥20
High sensitivity	≤5:1	≥20
High concentration	≥5:1	≥20

### In vivo animal studies

**Fig. 4:**
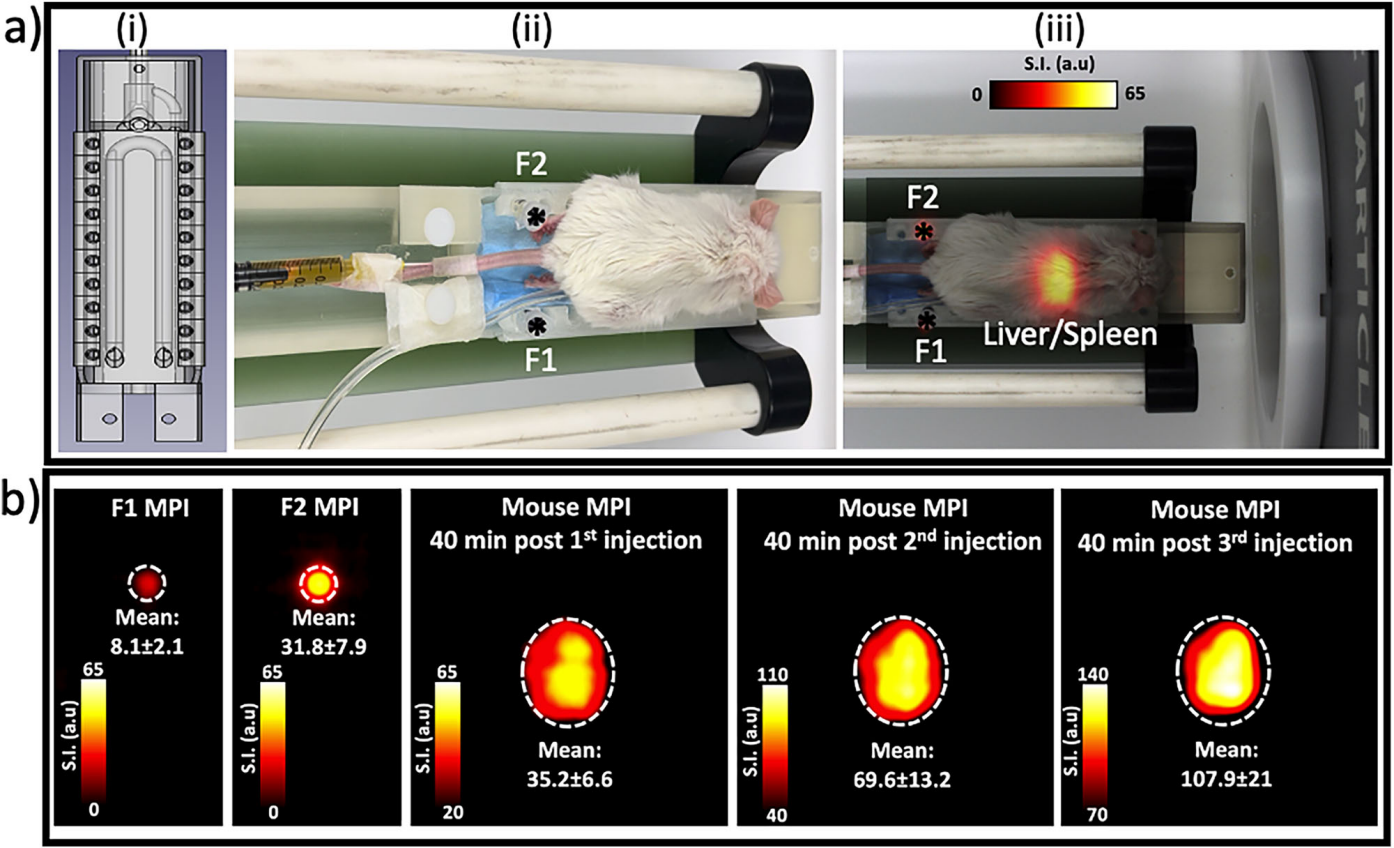
Experimental design and MPI signal quantification for fiducials and liver/spleen in a representative mouse. **a** (i) Design of a 3D-printed MPI bed for precision-aligned tracking of fiducials during repeated in vivo imaging. (ii) A mouse positioned on a customized MPI bed for repeated in vivo MPI sessions, with two distinct fiducials with precise alignment. The tail vein was catheterized for repeated injection and secured in place throughout the procedure. The syringe was filled with 400 µl of ferucarbotran at 200 µg Fe/ml. (iii) Representative MPI after the first injection. **b** MPI signal quantification of fiducials and the liver/spleen at 40-min intervals following sequential injections, free from any other hotspot interference. The first two injections contained 20 µg of iron, while the third dose doubled to 40 µg, totaling 80 µg of iron.

For in vivo imaging experiments, it is essential to use an MPI scan mode that is rapid enough for serial dynamic imaging while minimizing the severity of the spillover effect. Our phantom studies showed that both the standard and HS modes have comparable data acquisition times (2.3 vs. 2.2 min) and a safe TFD (≥20 mm). We selected the standard mode for in vivo experiments due to its wider safety margin of TFCR values compared to the HS mode (≤10:1 vs. ≤5:1). The first of the three separate ferucarbotran injections delivered 20 µg Fe, followed by a 40-min period interval to allow for magnetic tracer accumulation and signal saturation in the liver and spleen. Figure 4a-iii shows a distinct MPI signal in these two organs along with two faint fiducial signals. In Fig. 4b, the MPI signal from F2 is approximately four times stronger than that of F1, which aligns with the actual F2:F1 iron concentration ratio. The liver/spleen signal, measured 40 min after the second injection, is roughly twice that seen 40 min after the first injection, suggesting a proportional relationship between iron concentration and MPI signal intensity. However, the signal post-third injection does not show an expected doubling from the post-second injection or a quadrupling from the post-first injection, which may be due to a saturation limit of liver Kupffer cell and spleen macrophage MNP uptake.

**Fig. 5:**
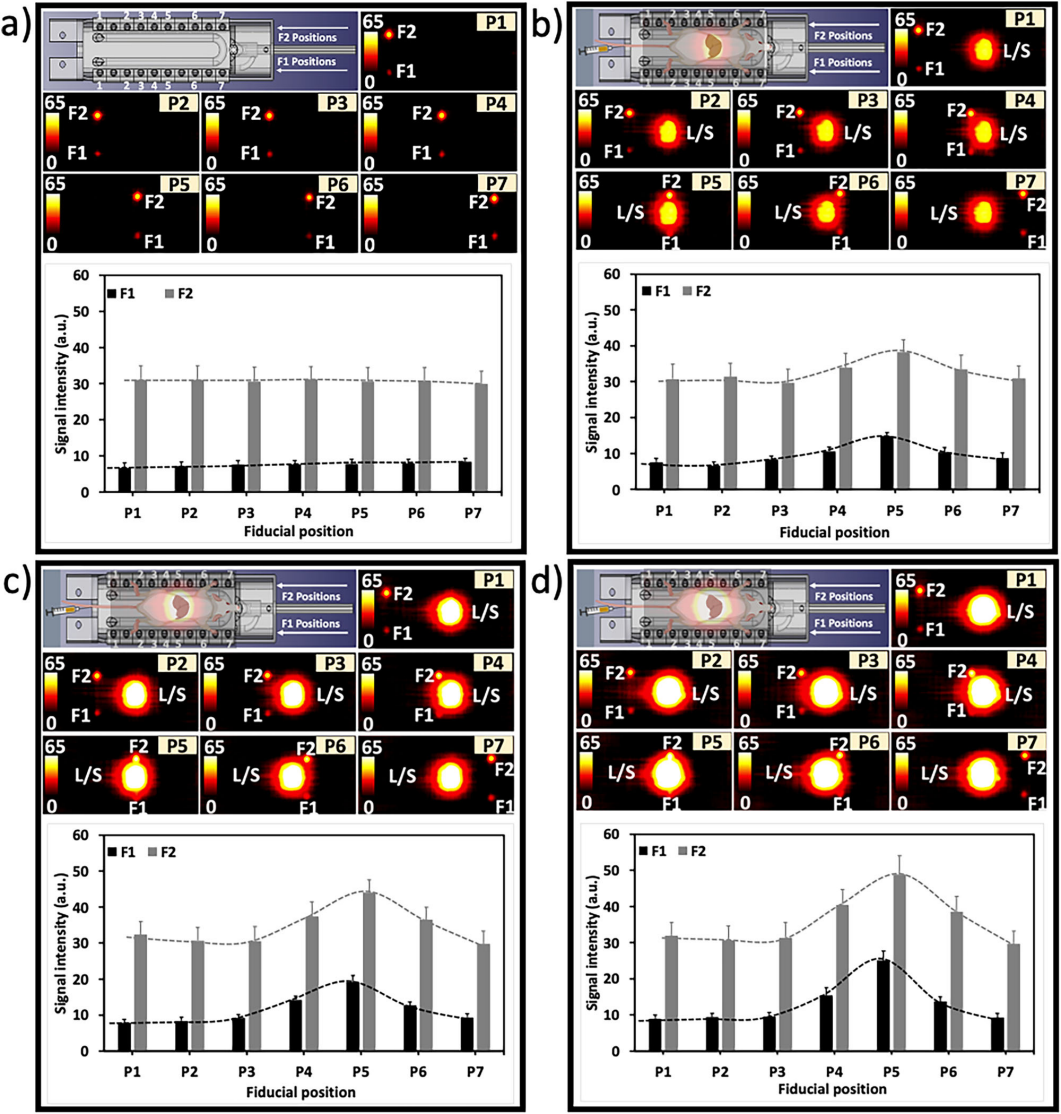
The spillover effect on fiducial signals during dynamic in vivo MPI. Top panels present the experimental setup and MPI scans of fiducials F1 and F2 placed at positions P1-P7 near the liver/spleen (L/S), **a** with no mouse and **b**–**d** post intravenous sequential injection of **b** the first 20 µg, **c** the second 20 µg, and finally **d** the additional 40 µg iron. Images were obtained 40 min after each injection. Bar graphs represent quantification of signal intensity for each fiducial, showing how the spillover effect impacts the accuracy of signal measurement as a function of the proximity of fiducials to a tissue of interest. Error bars represent the SD of the mean signal intensity, measured in pixels within the ROIs.

When the mouse was not placed within the FOV, the MPI of isolated fiducials demonstrated no significant signal intensity variations as their positions were altered (Fig. 5a). In the presence of the mouse, post-injection analyses showed distinct signal enhancements at varying fiducial positions (Fig. 5b–d). After the first injection, signal intensities at position P4 saw an increase of 14% for F2 and 28% for F1 compared to P3. Position P5 demonstrated a 29% increase for F2 and 80% for F1, while the transition from P6 to P7 revealed signal reductions of 8% for F2 and 18% for F1. Following the second injection, the increment from P3 to P4 was 22% for F2 and 55% for F1, and from P3 to P5, it was 43% for F2 and 109% for F1. From P6 to P7, decreases were 22% for F2 and 35% for F1. After the third injection, changes from P3 to P4 showed a 30% rise for F2 and 62% for F1, and from P3 to P5, a 55% increase for F2 and 164% for F1 were observed. The movement from P6 to P7 resulted in 30% lower signals for F2 and 47% for F1. The consistent increase in signal intensities for fiducials at the same position with each subsequent injection dose indicates that the MPI signal spillover effect is dose-dependent. The in vivo findings are in good agreement with the in vitro phantom studies, which can be expected given that MPI directly detects the magnetic signal from the magnetic tracer^32,33^. However, this relationship is not perfectly linear, particularly in an in vivo MPI setup. This fact was evidenced by the less-than-expected increase in liver/spleen signal after the third injection (Fig. 4b). Moreover, fiducial signal variations indicate a significant impact of fiducial positioning on signal detection. It became apparent that as the spacing between fiducials and the liver and spleen widens, the variation in measured signals for fiducials decreased. This implies that the proximity of liver/spleen and fiducials can significantly affect the accuracy of MPI signal quantification.

## Discussion

MPI uses a spatially fluctuating magnetic field from 2 to 8 T/m with selective magnetic saturation of MNPs within an FOV^32,34^. For the standard, HR, and HC scanning modes, a gradient of 5.7 T/m is used; for the HS mode this value is 3 T/m. At the origin of the gradient, a field free region (FFR) is present, characterized by a magnetic field strength that is weaker than the saturation values of MNPs^33^. Although it is expected that MNPs outside the FFR are magnetically saturated and generate no signal, our experimental observations demonstrate that real-world conditions deviate from this ideal scenario. It has been reported that ferucarbotran generates a signal akin to a 17 nm diameter magnetic iron oxide core, resulting in a native resolution of about 3.5 mm for a 2.35 T/m gradient^35,36^. Increasing the gradient to 6 T/m suggests the FFR is a millimeter-scale spheroid^34^, but our data indicate this scale is unreliable for accurately quantitative MPI studies.

Enhancing MPI spatial resolution has the potential to mitigate the spillover effect by rendering the boundaries between various ROIs more distinct, thereby reducing signal leakage between different spots. As described in Eq. (1), the MPI resolution is fundamentally governed by the gradient strength and MNP properties^34^:

1 $$\varDelta x\propto \frac{T}{{M}_{Sat}{d}^{3}G}$$

where *Δx* is spatial resolution, *T* is the temperature, *M*_*Sat*_ is the MNP saturation magnetization, *d* is the MNP diameter, and *G* is the gradient field strength. Collaborative efforts between chemists and engineers are essential to improve MPI spatial resolution, ultimately enhancing quantification strength and reliability of MPI technique by minimizing the spillover effect. Electrical engineers are actively working on the development of next-generation MPI scanners, targeting a gradient strength of 10 T/m. Such systems are anticipated to achieve sub-millimeter resolution without requiring modifications to the existing tracer^34^. However, magnetic colloid chemistry advancements may lead to improvements of MNP formulations with an improved MPI spatial resolution. Using engineered MNP tracers with higher spatial resolution^31^ may mitigate the MPI spillover effect because their signal does not spread as far and thus is less likely to interfere with other nearby sources in the FOV. It has been reported that rod-shaped MNPs can provide MPI images with improved spatial resolution compared to spherical MNPs^37^. Studies also suggest that particles with low magnetic anisotropies exhibit superior MPI performance compared to those with higher anisotropies^38^. According to Eq. (1), a 25% increase in MNP diameter from 17 to 22 nm can result in a doubling of spatial resolution Improving MPI spatial resolution may also be achieved by enhancing the MNP saturation magnetization. Theoretically, based on Eq. (1), temperature should affect MPI spatial resolution, although changing temperature may not be a practical approach in clinical settings. However, temperature changes may become a concern for signal quantification during an MPI-guided hyperthermia procedure^10^, where MPI data are used for quantitative guidance. According to Eq. (1), when a tumor loaded with MNPs is heated, the spatial resolution should decrease, potentially increasing the spillover effect and leading to unreliable signal quantification. However, in our recent study^39^, we showed that while the MPI signal intensity of ferucarbotran decreases with increasing sample temperature, the change in spatial resolution with temperature was negligible.

It would be of interest to compare field-free line and field-free point-based scanners to determine the extent of the spillover effect for each acquisition method. Software engineers have the potential to improve MPI spatial resolution by refining the two primary image reconstruction techniques employed in MPI: harmonic-space MPI and x-space MPI^34^. Since MPI shares many similarities with nuclear imaging methods^22^, where the PVE can lead to signal under- or over-quantification due to spillover from limited spatial resolution, strategies developed to address PVE in nuclear imaging may also be effectively adapted to mitigate spillover effects in MPI. For example, the application of deep learning^40,41^ and machine learning^42^ techniques have significantly contributed to improved spatial resolution and a reduction in the spillover effect to enhance the overall performance and accuracy of MPI.

It is essential to recognize and mitigate the spillover effect in order to improve the accuracy and reliability of MPI as a quantitative imaging technique. The existence of the spillover effect does not necessarily imply that a reliable MNP quantitative imaging cannot be achieved, rather, it suggests that careful optimization and calibration of the MPI procedures is essential. With the current use of pre-clinical MPI scanners, the fiducial signal remains the only reference for quantifying MPI hot spot(s) within a living subject. For reliable and standardized in vivo dynamic quantification, the fiducial signal(s) must maintain consistency across all experimental conditions. This requires that the fiducial signal(s) should not vary over time. By following the recommendations outlined in this paper regarding TFCR, TFD, and MPI scan mode, more consistent signal quantification for fiducials may be achieved, even in dynamic scenarios. MPI scanner manufacturers and magnetic tracer developers are encouraged to devise methods to minimize the spillover effect in living subjects as the actual distances between signal sources will vary and are often not known a priori.

## Methods

### In vitro phantom studies

Ferucarbotran (a carboxydextran-coated iron oxide and the active pharmaceutical ingredient in Resovist®, stock concentration 1 M Fe) was purchased from Meito Sangyo Co. (Nagoya, Japan) and used as MPI tracer throughout our studies. Two sets of high and low ferucarbotran concentrations were prepared. The high concentration samples acted as tissue mimicking phantoms “target tubes”), while the low concentration samples served as calibration fiducials. The position of target tubes remained constant throughout the MPI experiments, while the fiducials were moved to assess the impact of varying TFD setups. Target tubes contained 200, 400, and 800 µg Fe/ml in a volume of 100 µl 10 mM PBS, pH = 7.4. Fiducial tubes containing 10, 40, and 160 µg Fe/ml were also prepared with the same volume. Four replicates were made for each fiducial Fe concentration. 2D MPI was performed using a Momentum MPI scanner (Magnetic Insight, Alameda, CA). From each set, a tube with either the median signal intensity or the most frequently occurring MPI signal intensity was selected for subsequent experiments in the presence of a target tube. A custom MPI bed with two side racks equipped with holes for securing fiducials was designed using FreeCAD 0.21.0 and then manufactured with a 3D printer (Ultimaker 2 Extended+). In the printed bed, the center of one hole was positioned 10 mm away from the center of the adjacent hole. We tested four TFD values ranging from 10 to 40 mm in 10 mm increments. For consistency, the same MPI bed was used for evaluation of the four available MPI scan modes of Momentum MPI scanner, i.e., standard, HR, HS, and HC. Different MPI scan modes are installed by the vendor as options for optimal MPI scanning, i.e., detecting low signals at the cost of resolution, achieving higher resolution at the expense of acquisition time, or quantifying high iron levels. All imaging was performed with a Z center at 30 mm and a Z field of view at 12 cm, with image acquisition times of 2.3, 4.5, 2.2, and 5.5 min for standard, HR, HS, and HC modes, respectively. The Momentum MPI scanner software can provide two types of scans: a “native” scan with minimally processed data and a “sharpened” scan that applies further processing via deconvolution. Recent research has shown that signals spread farther from an SPIO source in “native” images^22^. Therefore, “sharpened” images were analyzed using ImageJ software, with consistent windowing parameters maintained for images acquired under identical scan modes throughout our studies. By adjusting minimum, maximum, brightness, and contrast of the images, windowing parameters were fine-tuned to avoid blooming effects from high Fe concentration hot spots in each scan mode. Using the ROI manager tool in ImageJ, consistent ROI areas were maintained across all image analyses.

### In vivo animal studies

All animal experiments were conducted in accordance with the Animal Welfare Act, the Public Health Service Policy on Humane Care and Use of Laboratory Animals, and relevant Johns Hopkins University guidelines. This study protocol was reviewed and approved by the JHU Institutional Animal Care and Use Committee. Male immunodeficient Rag2^−/−^ mice (6–8 weeks old; the Jackson Laboratory) were housed in standard cages within a specific pathogen-free facility. The environment was maintained on a 12-h light/dark cycle, at a temperature of 20–22 °C, and humidity levels of 40–60%. Mice had unrestricted access to standard laboratory chow and autoclaved tap water. The in vivo study involved intravenous (i.v.) injection of ferucarbotran in three mice. Given the exploratory nature and small sample size, formal randomization and blinding were not performed. For both injection and imaging procedures, mice were anesthetized using isoflurane inhalation. Anesthesia was induced with 3% isoflurane and maintained at 1.5–2% via a nose cone, ensuring the animals remained unconscious and immobile throughout the procedures. No analgesics were administered, as the procedures involved only minor interventions (i.v. injections and imaging) that were not anticipated to cause significant pain or distress. I.v. injection was chosen for ferucarbotran administration, as this is the standard route for injecting Resovist® in a clinical setting^43^. When 2.0 mg Fe ferucarbotran is administered i.v. in rats, a steady state is attained within 10 min due to the ultrashort blood half-life with MNP uptake into the reticuloendothelial system (RES) of the liver, spleen and lymph nodes^1^. We therefore chose the liver and spleen for studying the spillover effect, as they represent the major ferucarbotran-accumulating organs being part of the reticuloendothelial system^1^. The MPI bed was scanned prior to placing the fiducials or mice to ensure it was free from any magnetic contamination. A flexible polymeric i.v. catheter (24 G, JELCO, FL) was inserted into the tail vein of Rag2 mice (*n* = 3) to allow for repeated injections. A syringe filled with 80 µg Fe in 400 µl PBS was attached to the catheter for injections. The first of three separate ferucarbotran injections delivered 20 µg Fe (100 µl), followed by a 40-min period for complete uptake and signal saturation in the liver and spleen. Two fiducials containing 1 µg and 4 µg Fe in 100 µl PBS were then placed in different positions near the two organs and their signals were measured using a 2D MPI scan in standard mode, both within the same FOV and in separate FOVs. The standard mode was chosen for its rapid image acquisition, suitable for serial imaging in fast dynamic in vivo studies. In the initial phase of our studies, the phantom experiments demonstrated that the standard scan mode exhibited a notable advantage in visualizing multiple hot spots within the FOV at differing Fe concentrations. After the first injection and all image acquisitions, the whole process was repeated after two more injections. For the second injection, we administered 20 µg Fe in 100 µl PBS and for the third injection, we doubled the dose to 40 µg Fe in 200 µl PBS, both taken from the syringe used for the first injection. After each injection, a 40 min waiting interval before imaging was implemented to assure complete saturation of liver and spleen uptake. Following endpoint, mice were euthanized using CO₂ inhalation followed by cervical dislocation, in accordance with the American Veterinary Medical Association Guidelines for the Euthanasia of Animals. Sharpened images from in vivo experiments were created using ImageJ software similar to the approach described for the in vitro phantom studies.

## Data Availability

The datasets are available from the corresponding author on reasonable request.
